# Candida esophagitis incidentally detected by 18F‐FDG PET/CT in a patient with CNS lymphoma

**DOI:** 10.1002/ccr3.4322

**Published:** 2021-05-24

**Authors:** Melikşah Bayar, Nurgül Özgür Yurttaş, Seçkin Bilgiç, Kebire Karakuş, Uğur Önal, Kerim Sönmezoğlu, Zafer Başlar, Ahmet Emre Eşkazan

**Affiliations:** ^1^ Department of Internal Medicine Cerrahpaşa Faculty of Medicine Istanbul University‐Cerrahpaşa Istanbul Turkey; ^2^ Division of Hematology Department of Internal Medicine Cerrahpaşa Faculty of Medicine Istanbul University‐Cerrahpaşa Istanbul Turkey; ^3^ Department of Nuclear Medicine Cerrahpaşa Faculty of Medicine Istanbul University‐Cerrahpaşa Istanbul Turkey; ^4^ Division of Gastroenterology Department of Internal Medicine Cerrahpaşa Faculty of Medicine Istanbul University‐Cerrahpaşa Istanbul Turkey

**Keywords:** 18F‐FDG PET/CT, candida, endoscopy, esophagitis, lymphoma

## Abstract

Corticosteroids are commonly used in lymphoma patients, and findings in favor of esophageal involvement in 18F‐FDG PET‐CT should be considered suspicious and definitely be confirmed by biopsy. We describe a 58‐year‐old lady with diffuse large B‐cell lymphoma and central nervous system involvement having an increased metabolism in the distal esophagus with 18F‐FDG PET/CT, which was consistent with esophageal candidiasis, most probably due to prolonged use of dexamethasone. Esophageal candidiasis can be misdiagnosed as malignancy with a high SUVmax and may lead to difficulties while managing these patients.

## Graphical abstract

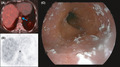

A 58‐year‐old woman was evaluated due to dizziness and unsteady gait. Brain contrast‐enhanced computed tomography (CT) showed a mass in the left occipital lobe. Excisional biopsy revealed diffuse large B‐cell lymphoma. An 18F‐FDG positron emission tomography/CT (PET/CT) scan for staging showed increased metabolism in the distal esophagus and left tonsil other than the occipital mass (Figure 1A‐E and Figure 2A,B). Prior to PET/CT, she received dexamethasone 12 mg/day for 50 days, and she had no dysphagia or odynophagia at the time of PET/CT. The upper gastrointestinal endoscopy showed multiple raised white plaques throughout the esophagus consistent with the benign etiology—esophageal candidiasis—in contrast to high SUVmax in 18F‐FDG PET/CT (Figure 2C). Oral 200 mg/day fluconazole was started, and the biopsy confirmed *Candida albicans* esophagitis.

**FIGURE 1:**
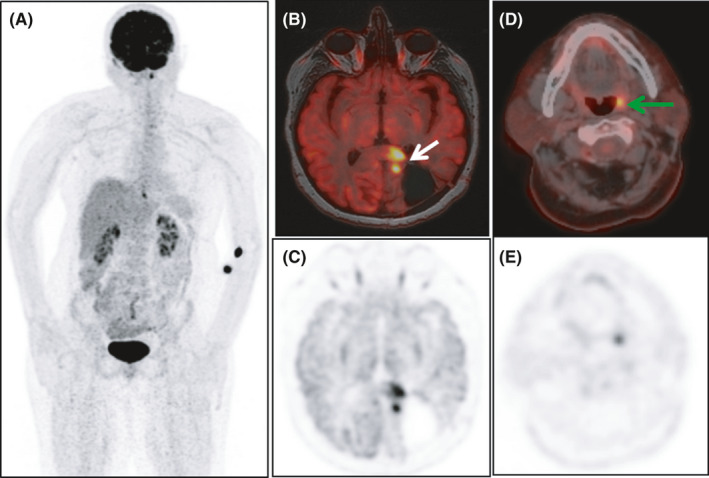
To examine the spread of disease, whole‐body 18F‐FDG PET/CT and brain PET/MRI were performed. The study revealed hypermetabolic lesions in the left occipital lobe, left tonsil, and distal esophagus in maximum intensity projection (MIP) images (A). 18F‐FDG PET/MRI images showed multiple intense hypermetabolic lesions (SUVmax: 22.75) adjacent to the operative defective area extending from the left occipital lobe level to the parietal lobe in the fused axial PET/MRI (B, C—solid arrow). Asymmetrical uptake of FDG (SUVmax: 9.68) demonstrated by the arrow on the axial 18F‐FDG PET/CT images localizes to the left tonsil (D, E—solid arrow)

**FIGURE 2:**
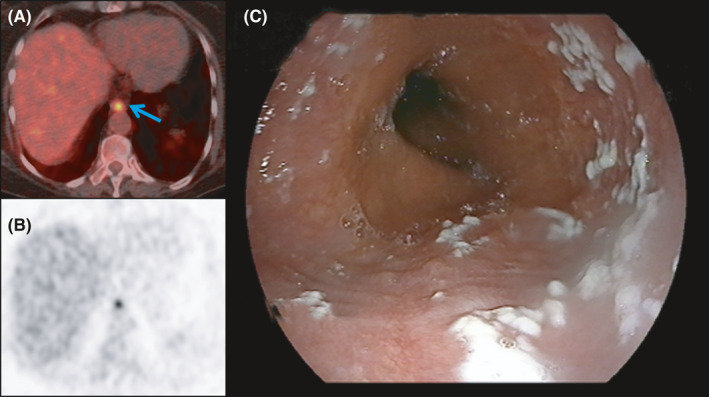
Axial 18F‐FDG PET/CT images showed focal 18F‐FDG uptake in the distal esophagus (SUVmax: 8.68), suggesting an etiology that may have clinical significance (A—solid arrow and B). Upper endoscopy demonstrated multiple whitish plaques corresponding to focal FDG uptake in the distal esophagus (C)

Esophageal FDG uptake is multifactorial, and both malignant and nonmalignant conditions may cause abnormal FDG uptake including esophageal candidiasis.^1^ Unexpected visualization of esophageal FDG uptake should be evaluated by endoscopy especially if the SUVmax of the lesions is greater than 3.5.^2^ *Candida albicans* infection is often observed in immunocompromised patients, and most probably, prolonged administration of corticosteroids due to the intracranial mass predisposed the generation of esophageal candidiasis in our patient.

## CONFLICT OF INTEREST

All authors have no conflict of interest to declare.

## AUTHOR CONTRIBUTIONS

MB, NÖY, SB, KK, and UÖ: wrote the paper. KS, ZB, and AEE: edited the paper. All authors approved the final draft.

## ETHICAL APPROVAL

A written informed consent was gathered from the patient.

## Data Availability

The data that support the findings of this study are available from the corresponding author upon reasonable request.
